# Quantitative Mass Spectrometric Analysis and Post-Extraction Stability Assessment of the Euglenoid Toxin Euglenophycin

**DOI:** 10.3390/toxins5091587

**Published:** 2013-09-18

**Authors:** Danielle B. Gutierrez, Alexandra Rafalski, Kevin Beauchesne, Peter D. Moeller, Richard E. Triemer, Paul V. Zimba

**Affiliations:** 1 Center for Coastal Studies Texas A&M University Corpus Christi 6300 Ocean Drive Corpus Christi, TX 78412, USA; E-Mail: dgutierrez@tamucc.edu; 2 Fisheries and Wildlife, Michigan State University, East Lansing, MI 48824, USA; E-Mail: rafalsk2@msu.edu; 3 NOAA/NCCOS Center for Human Health Research Hollings Marine Laboratory, 331 Fort Johnson Rd, Charleston, SC 29412, USA; E-Mails: kevin.beauchesne@noaa.gov (K.B.); peter.moeller@noaa.gov (P.D.M.); 4 Department of Plant Biology, Michigan State University, East Lansing, MI 48824, USA; E-Mail: triemer@msu.edu

**Keywords:** euglena, euglenophycin, stability, toxin

## Abstract

Euglenophycin is a recently discovered toxin produced by at least one species of euglenoid algae. The toxin has been responsible for several fish mortality events. To facilitate the identification and monitoring of euglenophycin in freshwater ponds, we have developed a specific mass spectrometric method for the identification and quantitation of euglenophycin. The post-extraction stability of the toxin was assessed under various conditions. Euglenophycin was most stable at room temperature. At 8 °C there was a small, but statistically significant, loss in toxin after one day. These methods and knowledge of the toxin’s stability will facilitate identification of the toxin as a causative agent in fish kills and determination of the toxin’s distribution in the organs of exposed fish.

## 1. Introduction

Freshwater algal toxins are produced by photosynthetic members of the cyanophyceae [1], dinophyceae [2,3], and euglenophyceae [4,5]. Toxins produced by these groups include neurotoxins, cytotoxins, dermal toxins, and a significant number with unknown targets. The impacts of harmful algal freshwater blooms include the direct impacts from fish kill events, the reduction in market valuation from decreased consumer confidence in fish products, and the ecosystem level changes in trophic structure through changes in light availability to benthic plants [6]. Additionally, harmful algal blooms negatively affect food resources, tourism and recreational water use [6]. 

The stability of algal toxins has largely been assessed in terms of stability once released into the environment or during drinking water treatment methods. Williams *et al.* (2008) evaluated the potential of selected biological toxins, primarily known dinoflagellate toxins, as biological attack weapons in chemical warfare and provided review of stability and treatment options for several of these compounds [7]. Microcystin has a half-life of 6.5–14 days once exposed to microbes [8]. Light and alkaline pH cause a rapid degradation of anatoxin-a [9], whereas light alone accelerates the decomposition of microcystins to form non-toxic compounds [10]. Additionally, the occupational and environmental health hazards of working with toxic cyanobacteria, from harvesting bulk material and mass culturing to purifying toxins were reviewed—use of appropriate safety clothing and equipment was recommended [11]. 

**Figure 1:**
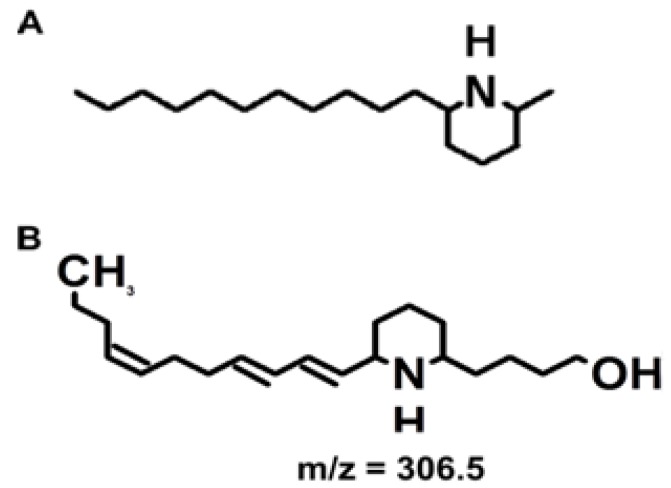
Toxin structures. (**A**) The structure of solenospin A, a component of fire ant venom. (**B**) The structure of euglenophycin. These structures were made with ACD/ChemSketch, Freeware version 12.01 (Advanced Chemistry Development, Inc. Toronto, ON, Canada).

In 2004, Zimba *et al.* reported the production of an icthyotoxin by a freshwater species of euglenoid, *Euglena sanguinea* Ehrenberg [4]. The toxin was first recognized after a fish mortality event in North Carolina and has since been the causative agent in more than 13 fish kills that totaled a loss of over $1 million. In the laboratory, fish exposed to cultured *E. sanguinea* cells and filtrate displayed altered behavior including disorientation and loss of equilibria [4]. Exposure to various concentrations of *E. sanguinea* cultures caused fish deaths within two hours [4]. The toxin structure was identified as a uniquely modified piperidine ring structure similar to the fire ant venom solenopsin [5]. Figure 1 shows the structure of the hydrated toxin (*m*/*z* of 306.5). The predominant ion detected by mass spectrometry analysis of biological samples and standards is the dehydrated toxin (*m*/*z* 288.3) [5]. Biological activity of euglenophycin was reported against other algal species, as well as inhibition of two cancerous tissue culture strains [5].

The environmental stability of euglenophycin and bloom treatment methods has not yet been assessed. As a first step, we have optimized a multiple reaction monitoring (MRM) method for specific analysis of euglenophycin and have determined the post-extraction stability of the toxin. Mass spectrometric analysis and knowledge of optimal handling procedures will facilitate identification and monitoring of euglenophycin as the causative agent in fish kills as well as future investigations, such as the toxin’s environmental stability and the distribution of toxin in the organs of exposed fish.

## 2. Results

### 2.1. Mass Spectrometric Analysis of Euglenophycin

**Figure 2:**
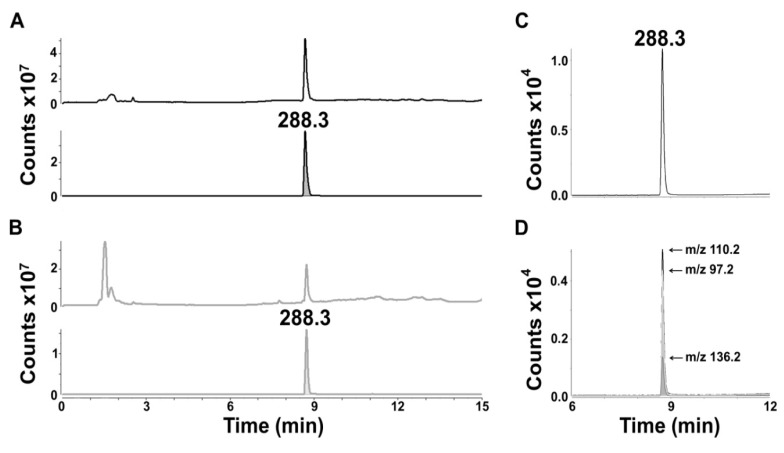
Mass spectrometric analysis of euglenophycin. Panels A and B show detection of the MH^+^-H_2_O ion of euglenophycin (*m*/*z* 288.3) from a MS scan (*m*/*z* 100–1000) of (**A**) purified euglenophycin (500 ng) and (**B**) euglenophycin extracted from a culture of *E. sanguinea*. Within each, the top panel shows the total ion chromatogram and the bottom panel shows the extracted ion chromatogram for *m*/*z* 288.3. Panels C and D show MRM detection of (**C**) purified euglenophycin at *m*/*z* 288.3 and (**D**) the monitored transition ions, *m*/*z* 110.2 (quantifier), *m*/*z* 136.2 and *m*/*z* 97.2.

Euglenophycin standards, extracted and purified from *E. sanguinea* cultures, were used to develop a specific MS/MS method for the identification and quantitation of the toxin in water samples. Figure 2A shows the full scan MS analysis of euglenophycin standard. The top panel shows the total ion chromatogram (TIC) and the bottom panel shows the extracted ion chromatogram (XIC) of the MH^+^-H_2_O ion of euglenophycin (*m*/*z* 288.3). The signal magnitude for the toxin in the XIC confirms the purity of the euglenophycin standard. For comparison, Figure 2B shows the full scan MS analysis of an extract from a toxin producing strain of *E. sanguinea*. 

In order to ensure specific detection of euglenophycin, a MRM method was developed. Figure 2C and Figure 2D show the detection of 1 ng of euglenophycin using this method. The transitions monitored were *m*/*z* 288.3 to *m*/*z* 110.2, 136.2 and 97.2. As shown in Figure 2D, *m*/*z* 110.2 was the most intense product ion; therefore, it was chosen as the quantifier ion. Throughout the analyses the qualifier/quantifier ion ratios were close to those obtained with the standards, typically differing from the standard average by less than 5%.

### 2.2. Post-Extraction Stability of Euglenophycin

**Figure 3:**
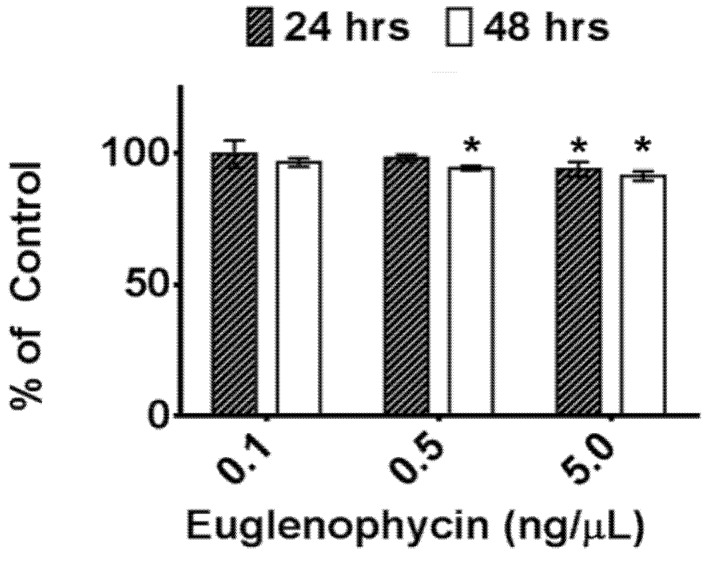
Euglenophycin stability at 8 °C. The stability of various concentrations (0.1, 0.5, and 5 ng/µL) of purified euglenophycin is shown after 24 h (striped) and 48 h (white) at 8 °C. Asterisks represent statistically significant differences from controls (see text). Error bars represent the standard deviation of two (24 h) or three (48 h) replicates.

The post-extraction stability of euglenophycin was assessed to determine optimal handling procedures during analysis. Euglenophycin standards were maintained at 8 °C (autosampler temperature) for 24 or 48 h prior to analysis. Figure 3 shows the amount of euglenophycin in each sample compared to the control (0 h at 8 °C) at various concentrations. At 5 ng/µL, a statistical difference was found between the control (100%) and both 24 h (93.8 ± 2.8%, *p* = 0.0053) and 48 h (91.3 ± 1.8%, *p* < 0.0001). At 0.5 ng/µL, a statistical difference was found between the control (100%) and the amount of euglenophycin at 48 h (94.3 ± 0.8%, *p* = 0.0042). At 0.1 ng/µL, no time points were significantly different from the control. 

**Figure 4:**
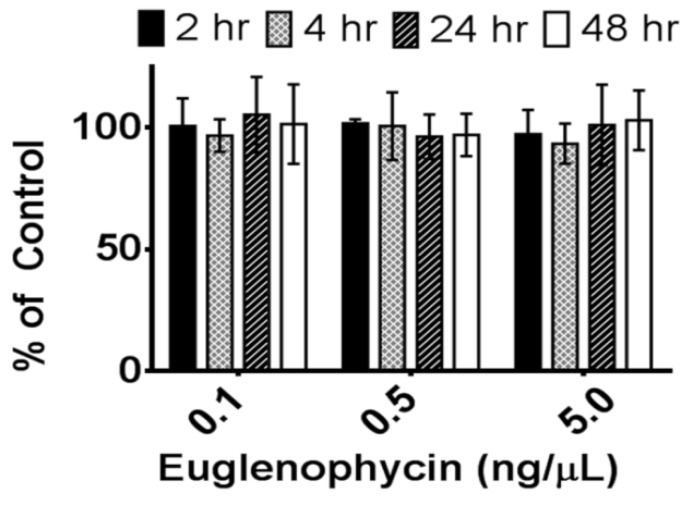
Euglenophycin stability at room temperature. The stability of various concentrations (0.1, 0.5, and 5 ng/µL) of purified euglenophycin is shown after 2 (black), 4 (checkered), 24 (striped), and 48 h (white) at room temperature. Error bars represent the standard deviation of three replicates.

The stability of euglenophycin at room temperature was assessed by storing samples at room temperature for 0, 2, 4, 24, or 48 h in the dark. Figure 4 shows the amount of euglenophycin in each sample compared to the control (0 h) at 0.1, 0.5 and 5 ng/µL. For all concentrations, there was no significant change in the amount of toxin over time at room temperature.

**Figure 5:**
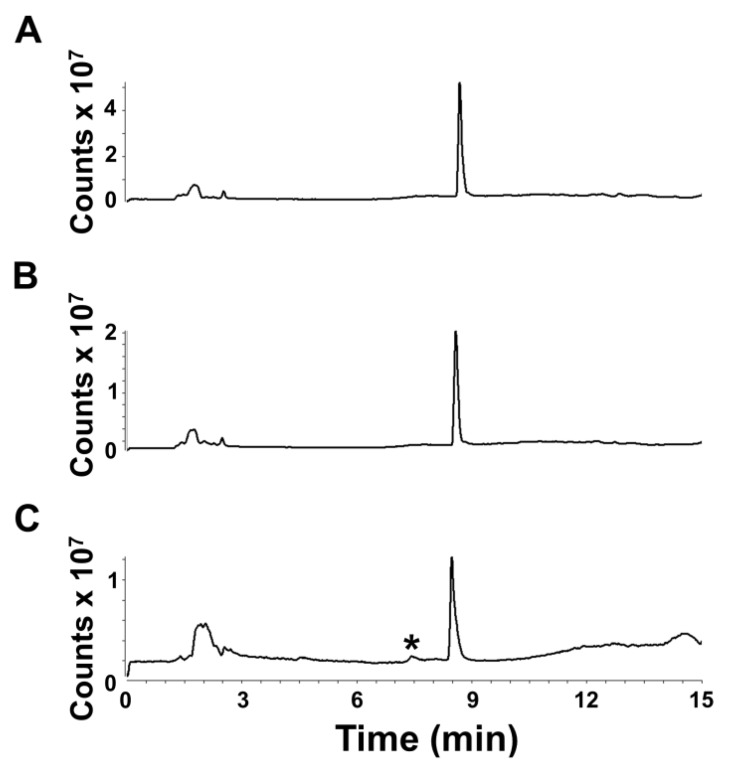
Long-term storage of euglenophycin. Total ion chromatograms from full scan analyses (*m*/*z* 100–1000) are shown for euglenophycin standards after (**A**) 1 day, (**B**) 38 and (**C**) 69 days of storage at −80 °C. The asterisk indicates a peak that was not detected after 1 and 38 days of storage at −80 °C.

Euglenophycin standards stored at −80 °C for up to one month did not show qualitative signs of degradation by full scan MS analysis (Figure 5A, one day, and Figure 5B, 38 days). The chromatograms have a similar appearance, with no obvious increase in degradation peaks over time. However, the total signal at 38 days decreased by 50% compared to day 1. After 69 days of storage at −80 °C, the total signal decreased by almost half again (Figure 5C).

Additionally, after 69 days of storage, a small peak appeared in the TIC eluting approximately 1 min prior to euglenophycin (Figure 5C, asterisk). The major ion eluting at this time was *m*/*z* 322.3. This peak was absent from the chromatograms of standard stored for 1 and 38 days at −80 °C, but in the chromatogram of standard stored for 69 days at −80 °C, it represented 4.7% of the combined peak areas for the euglenophycin and *m*/*z* 322.3 peaks. The relative amount of the *m*/*z* 322.3 ion in samples kept at 8 °C or room temperature for up to 48 h was similar compared to controls.

The effect of freezing at −80 °C and thawing on euglenophycin was also tested. Freezing and thawing standards three times did not significantly alter the amount of euglenophycin compared to controls (data not shown).

### 2.3. Analysis of Dissolved Euglenophycin

Zimba *et al.* (2004) reported that euglenophycin in the dissolved fraction of water samples was toxic to fish [4]. To investigate the presence of dissolved euglenophycin in water samples, we used euglenophycin standards to establish a solid phase extraction method for dissolved toxin analysis. Methanol elution of spiked *E. sanguinea* culture filtrates from a C_18_ solid phase extraction column resulted in detection of the toxin by MRM analysis; however, toxin recovery from spiked samples was typically less than 10% of the standard (data not shown). Dissolved toxin was also detected in two strains of *E. sanguinea* cultures when filtrates were passed through C_18_ solid phase extraction columns and eluted with methanol, 0.1% formic acid (data not shown). 

## 3. Discussion

Euglenophycin was first recognized after a fish mortality event in North Carolina and has since been the causative agent in more than 13 fish kills costing $1 million in lost revenue. Monitoring euglenophycin concentrations in field samples requires a quantitative method that is specific and that has high accuracy and precision. Furthermore, optimal handling is critical during the multistep procedures used for extracting and quantitating toxins from complex matrices [12,13]. 

In the present work, we have optimized our initial separation method for euglenophycin [5] to facilitate routine monitoring of water samples for the toxin in an unambiguous manner. We developed a MRM method for the identification and quantitation of euglenophycin. Identification of the toxin was based on three transitions, providing for specific detection of euglenophycin. Typically, the qualifier/quantifier ratios differed from the average standard ratio by less than 5%. 

Additionally, we assessed the post-extraction stability of euglenophycin to ensure optimal handling procedures. The data indicate that there is a statistically significant loss in toxin after one and two days at 8 °C at 5 ng/uL and after two days at 0.5 ng/uL. However, the actual differences were small: 6.1% ± 2.8% after 24 h and 8.7% ± 1.8% after 48 h for 5 ng/µL and 5.7% ± 0.8% after 24 h for 0.5 ng/µL. The data also show that euglenophycin is stable at room temperature with no statistically significant loss. The actual differences were less than 13% different from the control. The results suggest that euglenophycin is fairly stable for short time periods (1–2 days) at 8 °C and RT and that analyses should be kept at or below this length of time. While euglenophycin stored at −80 °C did not appear to have qualitative changes prior to two months, signal decreased steadily with each new batch analyzed. After two months of storage, signal continued to decrease and a small peak of *m*/*z* 322.3 appeared. This additional peak (shown in Figure 5C) could represent oxidized euglenophycin (addition of 16 Da to the parent compound). However, this peak only represents 4.7% of the total ion signal, and the decrease in euglenophycin signal was around 50%. The reason for the loss of euglenophycin signal at −80 °C is not apparent. At this temperature, the standards were stored at 1000 ng/µL. It is possible that the higher storage concentration provided conditions under which euglenophycin could self-react. One way to test this would be to store euglenophycin at −80 C at lower concentrations, such as 0.1, 0.5, and 5 ng/uL, and test if the loss in signal is reduced. 

Since standards were run prior to every experiment to create a standard curve and samples were compared to their controls, this loss in signal over time at −80 °C does not affect the results for euglenophycin stability at 8 °C and room temperature. However, it does suggest that standards need to be run prior to each analysis. Long term storage at room temperature may be an alternative way to maintain the standards, since the toxin appears to be stable at room temperature for short term storage; however, this has not been tested.

The mass spectrometric method developed here for specific and quantitative analysis of euglenophycin and knowledge of the toxin’s post-extraction stability are applicable to future investigations. This work will facilitate identification of euglenophycin as the causative agent in fish kills. It is a first step in developing methods for extracting, identifying, and quantitating euglenophycin present in fish and potentially other animals killed by toxin exposure. Additionally, these methods will be applied to assess various strains of *E. sanguinea* for toxin production in both cell bound and dissolved fractions. Continuing work on efficiently trapping the cell free toxin will be required.

## 4. Experimental Section

### 4.1. Euglena Culture and Purification

*E. sanguinea* Ehrenberg clonal cultures were isolated from several fish kill events in North Carolina and Texas [5]. Cultures were grown in AF6 media under 12:12 light:dark cycles at 28 °C. Cells were harvested in late exponential phase by centrifugation at 2000 rpm. Cell pellets from cultures were sonicated for 30 s in 1 to 2 volumes of 100% methanol and placed at 4 °C for 4–24 h. Euglenophycin was purified from the extract by high performance liquid chromatography (HPLC), as previously described [5]. Purified toxin was dried, weighed and stored at −80 °C. To make standards for quantitative analysis, the toxin was reconstituted in dimethyl sulfoxide to 1 µg/µL and stored at −80 °C under nitrogen.

### 4.2. Stability Studies

Euglenophycin standards were diluted to 0.1–50 ng/µL in 100% methanol and analyzed in triplicate to establish a standard curve (0.1–5 ng/µL) prior to each analysis for quantitation of toxin. To determine the effect of freezing and thawing, euglenophycin standards were stored at −80 °C for a minimum of 18 h and thawed to room temperature. This was repeated 3 times. Prior to analysis, the samples were diluted to 0.1, 0.5 and 5 ng/µL in 100% methanol. To assess the stability of euglenophycin at 8 °C, standards were diluted as above and either analyzed immediately or kept at 8 °C for 24 or 48 h prior to analysis. Similarly, to determine the stability of euglenophycin at room temperature, diluted standards were either analyzed immediately or placed at room temperature for 2, 4, 24, or 48 h prior to analysis. Toxin stability was assessed by comparing the amount of euglenophycin (as determined by the standard curve) in samples to the controls. All autosampler vials were pre-purged with nitrogen. 

### 4.3. Euglenophycin Extraction

Retentates from 50 mL of *E. sanguinea* culture were collected on Whatman GF/D glass microfiber filters (GE Healthcare Life Sciences, Piscataway, NJ, USA) and stored at −80 °C or processed immediately. Euglenophycin was extracted from the filters using 2 mL of methanol after a 4–24 h extraction period at 4 °C. The extracts were passed through 0.45 µm nylon syringe filters (Fisher Scientific, Pittsburgh, PA, USA) and transferred to autosampler vials pre-purged with nitrogen for liquid chromatography tandem mass spectrometry (LC-MS/MS) analysis.

### 4.4. Analysis of Dissolved Euglenophycin

Water was spiked with 1 mL of 1, 5, or 50 ng/µL of euglenophycin standard to a final volume of 15 mL. The samples were pulled via vacuum through preconditioned (charged using 3 mL of methanol then equilibrated with 6 mL of water) Strata C_18_-E, 3 mL × 100 mg solid phase extraction columns (Phenomenenex Corporation, Torrance, CA, USA). The columns were washed with 6 mL of water, and the analytes were slowly eluted with 1 mL of 100% methanol which was passed through the column a total of 3 times. 

### 4.5. Mass Spectrometry

LC-MS/MS analysis was carried out using Agilent MassHunter Data Acquisition software (version B.02.01) on an Agilent 1200 series HPLC in-line with an Agilent 6410 triple quadrupole mass spectrometer fitted with an electrospray ionization source. The samples were maintained at 8 °C using a thermostated autosampler and 10 µL were injected using the autosampler. The analytes were passed through a column shield prefilter (MAC-MOD Analytical, Inc., Chadds Ford, PA, USA) and loaded onto a Phenomenex Luna C_18_(2), 3 µm particle size, 150 × 3 mm column in 10% mobile phase A (90% water, 10% acetonitrile, 0.1% formic acid), 90% mobile phase B (100% acetonitrile, 0.1% formic acid) at a flow rate of 0.4 mL/min. Initial conditions were maintained for 2 min, and euglenophycin was eluted over a 6 min gradient from 10% to 90% mobile phase B followed by 3 min at 90% mobile phase B, before returning to initial conditions. 

Samples were analyzed in full scan (*m*/*z* 100–1000), positive ion mode for qualitative analysis and by MRM in positive ion mode for quantitation of euglenophycin. Agilent MassHunter Optimization software (version B.02.01) was used to determine the transition ions, optimal collision energies, and fragmentation voltages of the precursor ion (*m*/*z* 288.3). Transitions to the following product ions were monitored: *m*/*z* 110.2 (quantifier ion), *m*/*z* 136.2 and *m*/*z* 97.2. 

### 4.6. Data Analysis and Quantitation

Data were analyzed using Agilent MassHunter Qualitative Analysis software (version B.03.01). A standard curve (1/*y*^2^ weighting) was established by integrating the peak area of the quantifier ion from triplicate euglenophycin standards (6 concentrations ranging from 0.1 to 5 ng/µL). Integration was performed using MS/MS Integrator in the Qualitative Analysis software. To determine the amount of euglenophycin in each sample, the peak area of the quantifier ion was compared to the standard curve. For relative quantitation of ions in full scan mode, peak areas were integrated using MS Integrator in the qualitative analysis software and the relative amount of each was determined as a percent of the combined peak areas. Limit of detection was typically 0.3 ng/uL euglenophycin. To determine statistical differences in the amount of euglenophycin among controls and samples, two-factor ANOVAs were performed followed by Tukey’s multiple comparisons test (for samples stored at 8 °C and room temperature) or Bonferroni’s multiple comparisons test (for freeze-thaw samples) using GraphPad Prism 6.0 Demo (GraphPad Software, San Diego, CA, USA). 

## 5. Conclusions

Euglenophycin is a recently identified novel toxic compound, so baseline information on stability is lacking. The compound is stable at room temperature in the dark. When stored dry at −80 °C, it has a half life of about 30 days.
